# Laser Powder Bed Fusion of 25CrMo4 Steel: Effect of Process Parameters on Metallurgical and Mechanical Properties

**DOI:** 10.3390/ma18235390

**Published:** 2025-11-29

**Authors:** Agnieszka Kublińska, Damian Dzienniak, Maciej Sułowski, Jacek Cieślik, Piotr Ledwig, Kamil Cichocki, Paulina Lisiecka-Graca, Michał Bembenek

**Affiliations:** 1 Department of Manufacturing Systems, Faculty of Mechanical Engineering and Robotics, AGH University of Krakow, A. Mickiewicza 30, 30-059 Krakow, Poland; dzindziora@agh.edu.pl (A.K.); ddamian@agh.edu.pl (D.D.); 2 Department of Physical Metallurgy and Powder Metallurgy, Faculty of Metals Engineering and Industrial Computer Science, AGH University of Krakow, A. Mickiewicza 30, 30-059 Krakow, Poland; sulek@agh.edu.pl; 3 Department of Metal Forming and Metallurgical Engineering, Faculty of Metal Engineering and Industrial Computer Science, AGH University of Krakow, A. Mickiewicza 30, 30-059 Krakow, Poland; pledwig@agh.edu.pl (P.L.); cichocki@agh.edu.pl (K.C.); graca@agh.edu.pl (P.L.-G.)

**Keywords:** manufacturing, 3D printing, laser-based powder bed fusion of metals (PBF-LB/M), 25CrMo4 steel, AISI 4130 steel, volumetric energy density (VED), linear energy density (LED)

## Abstract

In this paper, the effects of 3D printing parameters on the metallurgical and mechanical properties of 3D-printed 25CrMo4 steel are presented. Using laser-based powder bed fusion of metals (PBF-LB/M), samples were fabricated under varying conditions of laser power, scan speed, and layer thickness. The study examined how variations in volumetric energy density (VED) and linear energy density (LED) influence the material’s performance. The results show a strong correlation between the printing parameters and key properties such as hardness, porosity, bending strength, compressive strength, and tensile strength. Appropriate VED and LED improved density, reduced defects, and enhanced mechanical performance, whereas excessive energy inputs introduced brittleness. These findings support the advancement of additive manufacturing technologies for high-strength steels and broaden their potential applications in the aerospace, automotive, and construction sectors.

## 1. Introduction

3D metal printing technologies, such as laser powder bed fusion of metals (PBF-LB/M) and electron beam melting (EBM), enable the fabrication of geometrically complex components that are difficult or impossible to produce using conventional manufacturing techniques [1,2]. These methods are increasingly applied in aerospace, automotive, medical, and energy sectors owing to their ability to produce lightweight, high-performance metallic parts [3,4]. In PBF-LB/M, components are manufactured layer-by-layer through selective melting of metallic powder with a laser in a protective gas atmosphere [5,6].

The 25CrMo4/AISI 4130 steel is a low-alloy structural steel characterized by high tensile strength, good ductility, and favorable fatigue resistance, making it suitable for load-bearing engineering and aerospace applications [7]. Additive manufacturing of 4130 steel offers advantages, such as reduced weight, improved design flexibility, and rapid prototyping, which are particularly important in the aerospace industry where mass reduction, mechanical performance, and time-to-production are critical [8,9,10,11,12,13].

Despite the growing use of LPBF for structural steels, only limited research has investigated how printing parameters affect the microstructure and mechanical properties of 25CrMo4 steel. Previous studies have focused mainly on powder preparation methods, process simulations, or general parameter optimization, but without establishing a comprehensive process–structure–property relationship for this alloy [14]. Recent work on other metallic systems, such as AlSi10Mg, 316L stainless steel, 410L steel, and Hastelloy X, demonstrates that mechanical performance and defect formation are highly sensitive to energy input, cooling rates, and melt-pool stability [15,16,17]. These findings highlight the need for material-specific parameter optimization in LPBF.

In LPBF-processed low-alloy steels, extremely high cooling rates typically generate a fine-grained microstructure and promote spontaneous martensitic transformation. The resulting microstructure is strongly influenced by thermal gradients in the melt pool: martensite forms predominantly at melt-pool boundaries, whereas bainite or tempered martensite can occur in regions exposed to lower cooling rates. Additionally, fine Mn- and Si-rich oxide dispersions have been reported in LPBF steels due to reactions between molten metal and residual oxygen in the processing atmosphere or on powder surface [18,19,20].

Although several recent studies have addressed the LPBF processing of structural and low-alloy steels, the available literature remains fragmented and does not provide a comprehensive understanding of how laser power, scanning speed, hatch spacing, and energy input collectively influence microstructural evolution, porosity formation, melt-pool stability, and mechanical response in 25CrMo4/AISI 4130 steel. Existing publications discuss general LPBF behavior, energy absorption mechanisms, or laser–powder interactions, yet none of these studies focus specifically on quenched-and-tempered low-alloy steels nor provide a systematic evaluation of their sensitivity to process parameters [21,22]. This highlights the need for an integrated experimental investigation linking LPBF processing conditions to microstructure, porosity, and mechanical performance for this class of materials. A similar process–structure–property relationship has been demonstrated in recent high-quality studies on advanced aluminum alloys produced via PBF-LB, where synergistic interactions between laser power, scanning speed, and hatch spacing were shown to govern melt-pool stability, defect formation, and grain refinement [23,24]. These works confirm that optimal mechanical properties arise not from maximizing the energy input but from precisely balancing the key processing parameters to ensure stable solidification and microstructural homogeneity [25].

The present study addresses this gap by providing the first comprehensive analysis of how the LPBF parameters affect the microstructural features, defect distribution, and mechanical properties of 25CrMo4/AISI 4130 steel, thereby supporting its potential use in advanced load-bearing engineering applications [26,27,28].

## 2. Materials and Methods

**Figure 1:**
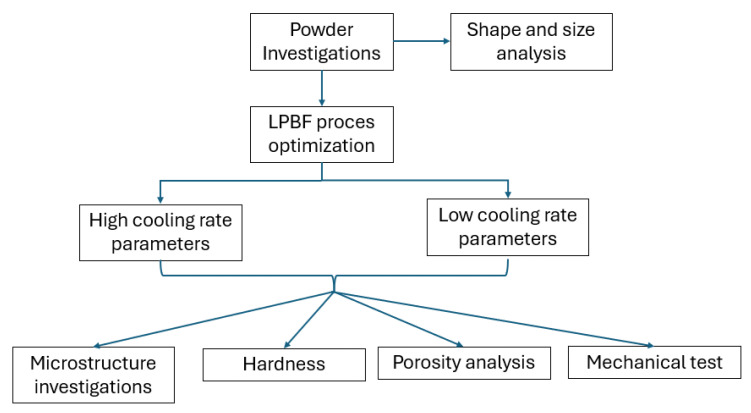
Overview of the methodological workflow used in the LPBF investigation.

The diagram in Figure 1 presents a comprehensive approach to analyzing the laser powder bed fusion (LPBF) process, starting from the powder characterization and culminating in the evaluation of material properties. The process begins with powder investigations, which are essential for ensuring the quality and consistency of the additive manufacturing feedstock. Within this stage, two key analyses were performed: shape and size analysis, which examine the morphology and granulometry of the metallic powder particles. These parameters play a role in determining the flowability and packing density of the powder, both of which significantly impact layer uniformity and the success of subsequent laser melting. Following this initial step, the process transitions into the adjustment of LPBF parameters aimed at reducing porosity and improving material integrity. Porosity analysis at this stage serves as a feedback mechanism, allowing engineers to assess the internal quality of printed parts and fine-tune the energy input, scan speed, and layer thickness accordingly.

Once the LPBF parameter set is defined, the strategy diverges into two main paths based on the cooling rates resulting from different processing conditions. At high cooling rate parameters, rapid solidification typically yields refined microstructures and potentially metastable phase formation. To evaluate these effects, both microstructure investigations and mechanical property testing were conducted. The former involves techniques such as optical microscopy, SEM, and EBSD to examine grain size, phase distribution, and defects. At the same time, the latter assesses the tensile strength, ductility, and other mechanical characteristics fundamental for functional applications.

In contrast, processing conditions that lead to lower cooling rates—allowing for slower heat dissipation and more extensive phase transformations—result in microstructures that are commonly assessed using hardness measurements. Hardness, which reflects both the microstructural state and the thermal history of the material, provides a rapid and informative indication of its resistance to deformation [16]. In this context, the terms “high” and “low” cooling rates refer only to relative differences between the tested parameter sets, not to absolute cooling-rate regimes.

The workflow in Figure 1 illustrates the interconnectedness of powder properties, processing conditions, and final material performance in the LPBF process. By systematically linking shape and size analysis to porosity outcomes and further correlating cooling rates to mechanical response, the diagram emphasizes a data-driven, iterative approach to refining process parameters in metal additive manufacturing [29].

### 2.1. Tested Material

The 25CrMo4 steel powder (Höganäs AB, Höganäs, Sweden) was used in this study. Its chemical composition is presented in Table 1.

**Figure 2:**
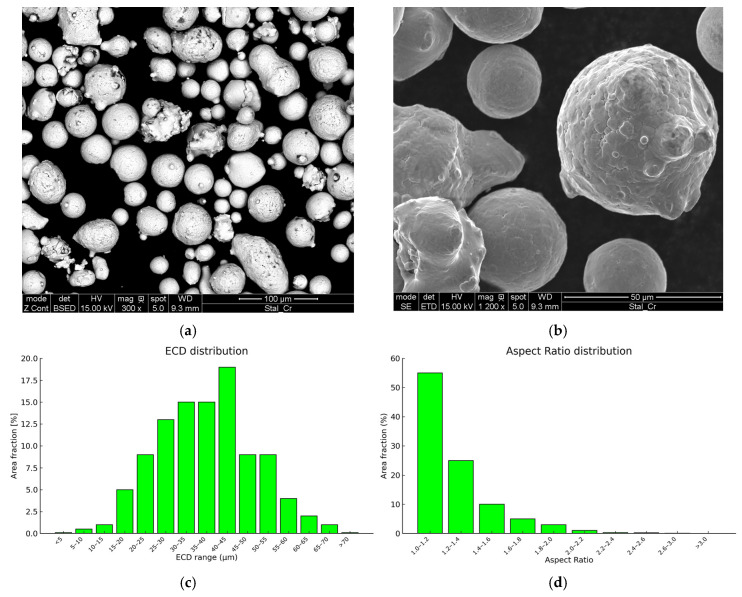
(**a**,**b**) SEM images of the 25CrMo4 powder; (**c**) ECD and (**d**) AR distribution histograms for the powder.

Figure 2 shows SEM images along with the results of the size and shape analysis of the 25CrMo4 powder. The powder was produced by gas atomization and exhibited a typical spherical morphology. The equivalent circular diameter (ECD) ranged from 5 to 70 µm. Particle size distribution followed a standard curve, with D10, D50, and D90 values of 25 µm, 41 µm, and 56 µm, respectively. In powder form, numerous shape defects were observed, including satellites, splat caps, broken, and elongated particles. However, overall, the powder was characterized by high sphericity, with a D50 aspect ratio (AR) of 1.175. Only 10% of the powder volume was characterized by particles with AR above 1.61. The powder was also characterized by a high flowability, which, combined with a suitable AR and particle distribution, made it suitable for LPBF [30].

Figure 2 presents the SEM images of the 25CrMo4 steel powder used in the study. Analyzing the shape and distribution of the powder particles is for assessing the quality of the material used in 3D printing. Most particles in the image are spherical, which is characteristic of high-quality powders used in LPBF technology. Spherical particles are desirable because they facilitate even distribution of the powder on the printing layer, reduce friction, and improve the powder’s flowability in feeding systems. Additionally, such particle shapes enhance sintering during the printing process, leading to reduced porosity in the final print [31].

Quantitative analysis was performed using ImageJ software (version 1.54g, National Institutes of Health, Bethesda, MD, USA) [32]. The AR was calculated as the ratio of the minimum and maximum Feret diameters, while the ECD was derived from the area of each particle. Size and shape analyses were conducted on five SEM images, covering a total area of approximately 540,000 µm^2^. Particle size was determined by manual contouring. Around 500 particles in the SEM images were analyzed.

### 2.2. LPBF Printing Procedure

**Figure 3:**
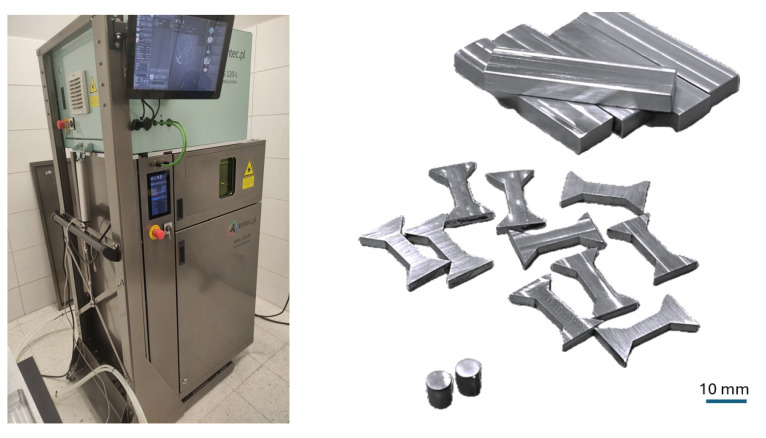
AYAS 120 ML LPBF machine and 25CrMo4 steel samples used in research.

The LPBF process was carried out using an AYAS 120 ML (Figure 3) 3D printer (Inntec, Gdansk, Poland). The 25CrMo4 steel build platform was preheated to 80 °C prior to processing. Powder melting was achieved using a laser with a maximum power of 200 W and a focal spot diameter of 35 µm. The process was conducted in an argon (Ar) atmosphere with an oxygen level maintained below 200 ppm [33].

The study was conducted in two stages. The first stage involved identifying a suitable process window by examining the porosity of cubes with 10 mm sides. All the samples were printed using a 30 µm layer thickness and two hatching distances: 70 µm and 90 µm. To determine the suitable LPBF parameters, various combinations of laser power and scanning speed were tested [34]. The process parameters used are summarized in Table 2.

Energy input was characterized using volumetric energy density (*VED*, Equation (1)) and linear energy density (*LED*, Equation (2)), which are key metrics for energy input in LPBF processes [35]. In this study, 30 samples were fabricated using various parameter sets [36]. The selection criterion required both qualitative and quantitative conditions to be met. Three of them were damaged during printing; therefore, so 27 samples were available for analysis. The results are shown in Table 2.

(1) $$VED\;=\;\frac{P}{v\;\cdot h\cdot t}$$

(2) $$LED=\frac{P}{v}$$

where

P—laser power [W];
v—scanning speed [mm/s];
h—layer thickness [mm];
t—scanning track width [mm].

The second stage involves selecting four process variants based on the print quality. The second stage involved mechanical testing and metallurgical analysis, which enabled the evaluation of the mechanical properties and microstructure of the samples produced using different process parameter variants.

Two hatch distances—70 µm and 90 µm—were selected as representative of typical industrial settings used in PBF-LB/M processing. These values correspond to a range comparable to the laser spot diameter and are commonly applied in the fabrication of alloy steels. As reported in the literature, excessively small hatch distances (<60 µm) tend to cause overheating and excessive remelting, whereas excessively large distances (>100–110 µm) increase the likelihood of lack-of-fusion defects. Therefore, 70 µm was adopted as a practical lower boundary ensuring sufficient track overlap, while 90 µm served as an upper boundary enabling the assessment of the effect of reduced overlap on melt pool stability. This selection allowed the experiment to cover both stable and potentially unstable melting conditions [37].

The laser power range (90–180 W) and scanning speeds (800–1200 mm/s) were chosen based on literature data and preliminary trials to avoid two undesirable phenomena: insufficient melting at low power levels (<90 W) and the formation of keyhole porosity or local overheating at powers exceeding 200 W. The selected values fall within ranges commonly used in industrial practice and lead to volumetric energy density (VED) values of approximately 30–100 J/mm^3^, which are widely recognized as suitable for steel processing in LPBF systems [38,39,40].

### 2.3. Quality and Porosity Type

The samples were cut in two planes: XY (parallel to the build layers) and XZ (perpendicular to the build layers) using a precision cutter. This allowed for the observation of grain structure and porosity in both the build direction and the cross-section. Subsequently, the specimens were embedded in a conductive resin to facilitate further processing during grinding and polishing. The preparation process followed a standard metallographic procedure: initial grinding was carried out using SiC abrasive papers with decreasing grit sizes, followed by polishing with diamond suspensions of 6 µm, 3 µm, and 1 µm. The final polishing step was performed using colloidal silica suspension (0.05 µm), which removed the deformed surface layer and produced a mirror-like finish. The prepared surfaces were then examined using an Inspect S50 microscope (FEI, Hillsboro, OR, USA). After that, photos of them were taken and analyzed. Based on the images, porosity and melting mode were classified into five categories: lack of fusion, low lack of fusion, process window, small keyhole, and keyhole.

The porosity of the samples was determined manually based on metallographic images taken after specimen preparation. The analysis involved visual identification and measurement of voids in the microstructure using ImageJ software, followed by calculation of the pore fraction in the material volume (expressed as a percentage). A manual approach was necessary because the available image-analysis software is not suitable for qualitative evaluation, particularly when assessing the presence and morphology of defects. For this reason, defects were identified and assessed manually to ensure reliable interpretation. Each measurement was performed in three repetitions, where possible, to increase the reliability of the results.

The porosity values obtained from the conducted analysis were used to define the process window in stage 1 of the study. According to literature on steels processed via the LPBF, porosity levels below approximately 0.2–0.3% are typically associated with stable melting conditions and are considered indicative of near-fully dense material [41,42]. At the same time, several studies report that porosity values reaching up to 0.35% may still be acceptable during preliminary parameter qualification, provided that the observed pores are not characteristic of lack-of-fusion defects and remain uniformly distributed within the microstructure. Based on these criteria, a practical porosity threshold of ~0.35% was adopted in this work when selecting the parameter sets for further mechanical and metallographic evaluation [43,44].

### 2.4. Hardness Test Procedure

The Innovatest Nexus (Innovatest, Maastricht, The Netherlands) is a hardness tester designed for Vickers hardness (HV) measurements, capable of applying a 0.5 kgf load. Each sample was tested for hardness 10 times. The average and standard deviations were taken from these measurements.

### 2.5. Tensile Tests Procedure

**Figure 4:**
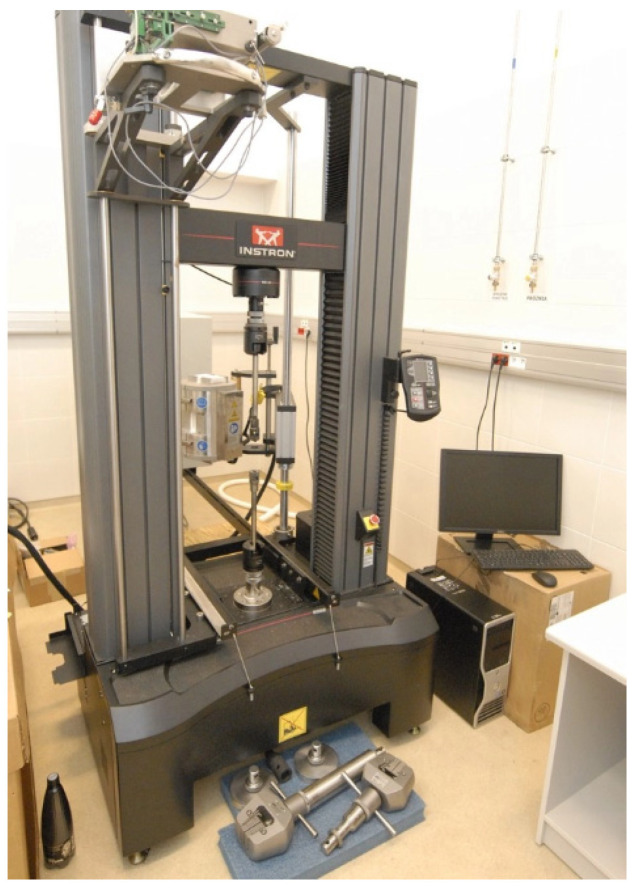
The testing machine Instron 5982.

**Figure 5:**
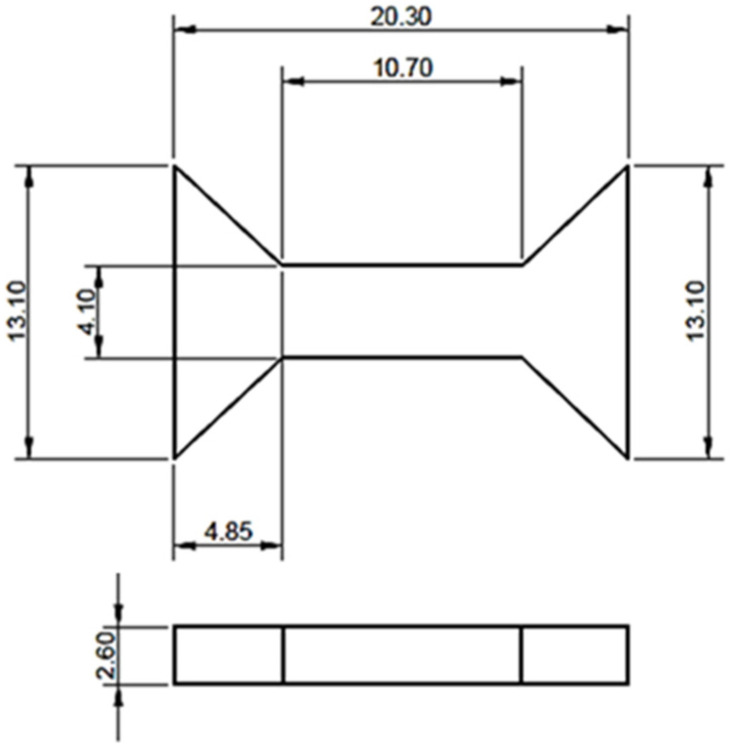
Tensile test specimen—technical drawing (unit: mm).

The tensile tests (Figure 4) were performed using an Instron 5982 universal testing machine equipped with the Bluehill^®^ 3 software (Instron Corporation, Norwood, MA, USA). Each series was tested using three samples in the tensile test. Dedicated grips were designed and prepared to ensure the proper fixation of the specimens during testing. The tests were conducted at a crosshead speed of 3 mm/min and repeated five times. The tensile specimens were not geometrically compliant with ISO 6892-1 [45] due to LPBF process constraints; however, the testing procedure (including strain rate) followed the standard recommendations. Figure 5 shows the tensile test samples with labeled dimensions.

### 2.6. Metallographic Tests Procedure

The Nova NanoSEM 450 (FEI, USA) scanning electron microscope was used for the study. It was equipped with a Schottky field emission gun (FEG), providing high-resolution imaging. The device allows operation in both high and low vacuum modes, enabling the analysis of both conductive and non-conductive samples without the need for metallization. The microscope was equipped with secondary electron (SE) and backscattered electron (BSE) detectors, and it was equipped with an electron backscattered diffraction (EBSD) EDAX detector by Gatan. The EBSD analysis was performed under a 70° sample tilt. The step size of EBSD map was equal to 0.1 µm, with accelerating voltage equal to 20 KV and spot 6.0. Grain size analysis was performed using the TSL-OIM™ Data Collection software [44,46].

### 2.7. Strain Test Procedure

The servo-hydraulic thermomechanical simulator ASP II by Servotest enables compression tests with a maximum force of ±100 kN and a displacement range of ±25 mm. The test was conducted under normal conditions. The compression test was performed on cylindrical specimens with dimensions Ø5 mm × 5 mm using a Servotest device. Each series was tested using three samples in the bending test.

### 2.8. Fracture Strength Test Procedure

The focus point bending tests were conducted using an Instron 4502 (Instron Corporation, USA) testing machine. The bending specimens had dimensions of 6 × 12 × 45 mm. In the three-point bending method, supports with a span of 28.6 mm and a loading roller with a diameter of 3.5 mm were used. The device had a maximum load capacity of 100 kN. During the tests, both the applied force and crosshead displacement were continuously recorded over time. The crosshead speed was set to 1 mm/min. These recorded data were then used to calculate the instantaneous stress and strain values [47,48].

(3) $$\sigma =\frac{3FL}{2bh^{2}}$$

where

F—applied bending force [N];
L—support span [mm];
b—specimen width [mm];
h—specimen height [mm];
D—specimen deflection at the midpoint [mm].

## 3. Results and Discussion

Below is a summary of the tests conducted on the 25CrMo4 material.

### 3.1. LPBF Process

In the first stage, porosity tests were carried out on a set of samples. In Table 3 and Table 4, the results of the influence of process parameters on porosity are presented. The samples 11, 30, 10, and 29 were selected for further investigation because they best represented the two key regions of the process. Samples 11 and 30 fell within the stable process window—they exhibited low porosity, no lack-of-fusion defects, and comparable LED values, making them representative of the suitable parameter range. Samples 10 and 29, on the other hand, were chosen as examples of higher-energy conditions in which the first signs of keyhole-type porosity appeared. This allows for assessing how the increased energy input affects the microstructure and mechanical properties. The remaining samples were excluded because they contained defects (e.g., numerous Lack of Fusions (LoF) and porosity > 1%), which prevented reliable analysis [49]. Therefore, four representative samples were selected to cover both stable and boundary LPBF processing conditions.

Porosity analysis revealed that for samples fabricated at a hatch distance of 70 µm, the process window (porosity below 0.2%) is contained for VED values of 65–77 J/mm^3^. For the lower VED range, defects such as lack of fusion begin to appear, while for VED values below 53 J/mm^3^, the porosity exceeds 0.5%. In contrast, for energies above this VED range, increasingly numerous keyhole pores appear; however, only for sample no. 13 (VED equal to 101 J/mm^3^) did the porosity reach a very high level [50].

The selection of process parameters in this study—specifically, hatch distances of 70 µm and 90 µm, laser power in the range of 90–180 W, and scanning speeds between 800 and 1200 mm/s—was based on both a review of relevant literature and the technological constraints characteristic of the Laser Powder Bed Fusion (LPBF) process.

The hatch distances of 70 µm and 90 µm were chosen as representatives of typical industrial practice, allowing for a balance between the effective powder melting and process efficiency. As reported in the literature, hatch distances below 60 µm often result in overheating and excessive material melting, whereas values above 100–110 µm significantly increase the risk of lack-of-fusion defects and porosity. Thus, 70 µm was adopted as a practical lower boundary for effective melting, and 90 µm as the upper limit suitable for evaluating the effect of reduced track overlap on melt pool stability and structural integrity [51].

The laser power range of 90–180 W was selected to avoid insufficient melting at the low energy levels (<90 W) and the formation of keyhole porosity or excessive overheating at high power values (>200 W). This range is commonly used in industrial LPBF processing of alloy steels and enables the achievement of high densification without compromising microstructural integrity [52,53].

Similarly, scanning speeds of 800–1200 mm/s were chosen to provide a realistic and representative range of volumetric energy density (30–100 J/mm^3^), commonly regarded as suitable for processing steel alloys. Lower scanning speeds promote heat accumulation and formation of residual stresses, whereas higher speeds increase the risk of incomplete melting [54,55]. Extreme parameter values (e.g., hatch distance < 60 µm, laser power > 200 W) were not included, as literature data and LPBF manufacturer guidelines clearly indicate that such settings lead to severe defects, such as extensive lack-of-fusion or unstable keyhole porosity. These conditions lie outside the practical industrial process window for steels such as 25CrMo4 and would not allow the fabrication of samples suitable for reliable mechanical testing.

Nevertheless, expanding the parameter window in future research could allow for a more precise identification of process boundaries and support the development of improved process adjustment strategies [53,54,55].

In summary, the applied process parameters reflect both the practical capabilities of LPBF systems and the research objectives. They enable a comprehensive analysis of how energy input affects the melt pool geometry, porosity, and mechanical performance, while operating within a safe and effective process window for 25CrMo4 steel.

**Figure 6:**
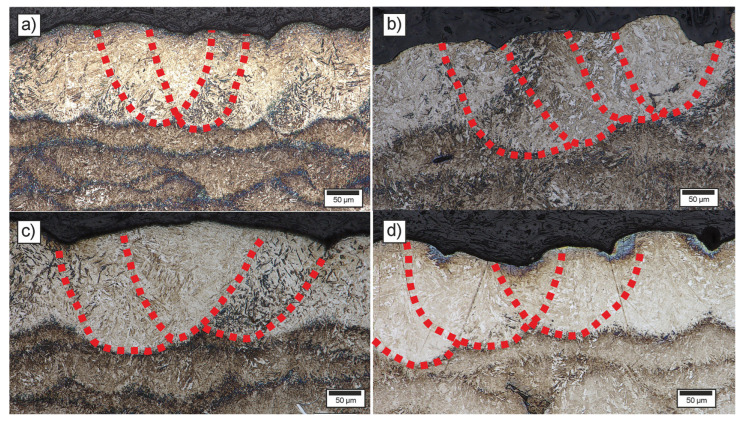
Microstructure of upper parts of LPBF-processed samples with marked melt pools boundaries for variant number (**a**) 10, (**b**) 11, (**c**) 29, and (**d**) 30.

The process window was more difficult to define for samples produced with a hatch distance of 90 µm, due to the larger melt pool volume and the need to balance lack-of-fusion defects at the track edges with overheating in the central region of the melt pool. In this case, the suitable VED values were in the range of 55–64 J/mm^3^, which is lower than the corresponding range identified for the 70 µm hatch distance. Additionally, the keyhole porosity formation was observed for higher VED parameters, while a lack of fusion was observed for lower energies. Additionally, the parameter region of accidentally lacking fusion porosity was wider than for 70 µm. In Figure 6, the microstructures of the upper parts of the samples were presented, including the melt pools’ boundaries. For parameter sets falling within the stable process window, two types of melt pools were observed. In the case of higher energy input (e.g., samples 10 and 29), corresponding to the keyhole melting regime, the melt pools were deeper and narrower. In contrast, for the lower LED values (e.g., samples 11 and 30), associated with the conduction or transition melting mode, the melt pools were wider and shallower [56]. The adjacent melt pools are shifted by the hatch distance.

The hatch distances were larger than the focal point of the laser beam, and the hatch disfocal point ratios were equal to 2.0 and 2.57. Therefore, wide and deep melt pools needed to be created to achieve the minimum porosity, which is why the conductive type of melt pools could not be achieved. The melt pool geometry was similar for the investigated materials, with a depth-to-width ratio of approximately 1–1.1 for samples with an LED of 0.18–0.19 J/mm^3^ (Samples 10 and 29). In contrast, for samples with an LED of 0.15 J/mm^3^ (Samples 11 and 30), the ratio was in the range of 0.65–0.8. The primary difference between samples differing in hatch distance was the degree of overlap in adjacent layers.

Based on the porosity results, four samples were selected for further analysis. Samples 11 and 30 were chosen because they fall within the process window and do not exhibit high porosity; lack of fusion-type porosity is absent, and their LED values are similar. Similarly, Samples 10 and 29 represent the range where the slight keyhole-type porosity occurs and have comparable LED values [57]. They were produced at higher energy levels, which leads to greater heat accumulation [58,59]. These results enabled the evaluation of how LPBF parameters and microstructure influence the mechanical behavior of 25CrMo4 steel, providing a foundation for improving the process and selecting appropriate parameter sets [60,61,62].

Following the initial qualitative assessment, which was crucial for eliminating defects, a more detailed quantitative porosity analysis was performed for four selected samples: 10, 11, 29, and 30. The porosity measurements (expressed as a percentage of volume) were conducted in three repeated trials, where applicable. The average porosity values obtained are presented in Table 5. The results confirm a clear difference in porosity levels, with samples 10 and 11 (which exhibited superior mechanical performance) showing lower or moderate porosity, while samples 29 and 30 were noticeably more porous.

**Figure 7:**
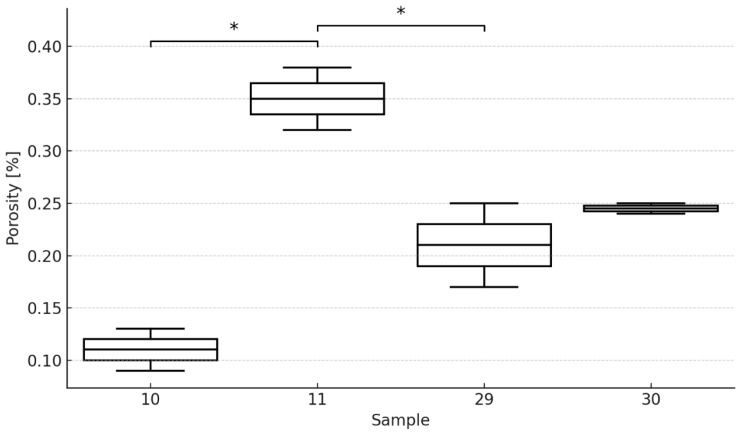
Comparison of porosity between samples using the one-way ANOVA.

The boxplot presents the porosity levels [%] of four investigated samples: 10, 11, 29, and 30 Figure 7. To evaluate statistical differences between the groups, a one-way ANOVA test was performed, followed by a Tukey post hoc test. The ANOVA results indicated statistically significant variation between the groups (F = 20.49, *p* = 0.0031), confirming that at least one sample differs significantly from the others in terms of porosity.

Subsequent analysis using Tukey’s test revealed that sample 11 exhibited significantly higher porosity compared to samples 10 (*p* = 0.004) and 29 (*p* = 0.006). These significant differences are marked with asterisks (*) on the plot. No statistically significant differences were found between the remaining groups.

Overall, the results suggest that process parameters used for sample 11 may have contributed to increased porosity, while samples 10 and 29 demonstrated more favorable, lower porosity values. The statistical analysis confirms that sample 11 stands out in terms of defect formation, which may impact its mechanical performance.

### 3.2. Microstructure Investigations

**Figure 8:**
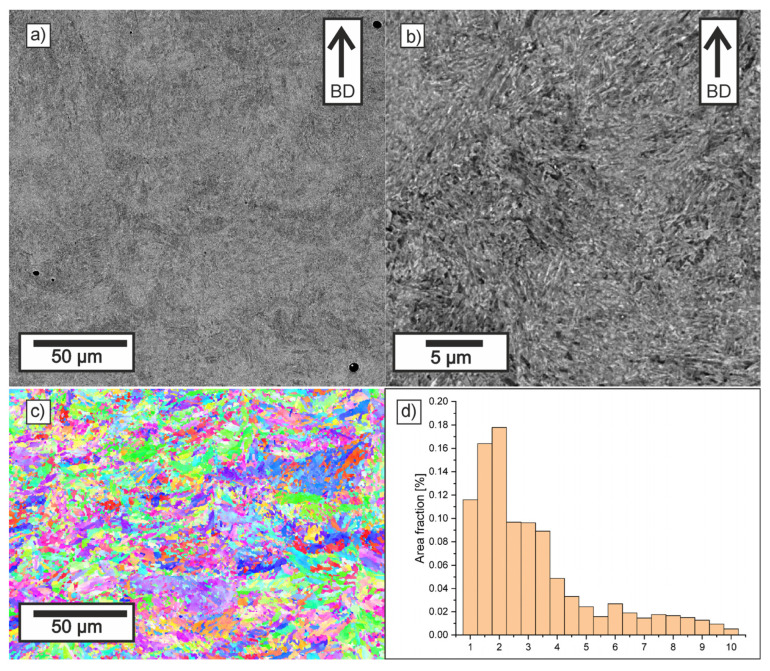
Microstructure of Sample 10: (**a**,**b**) SEM (BD-build direction), (**c**) EBSD IPF, and (**d**) grain size distribution.

**Figure 9:**
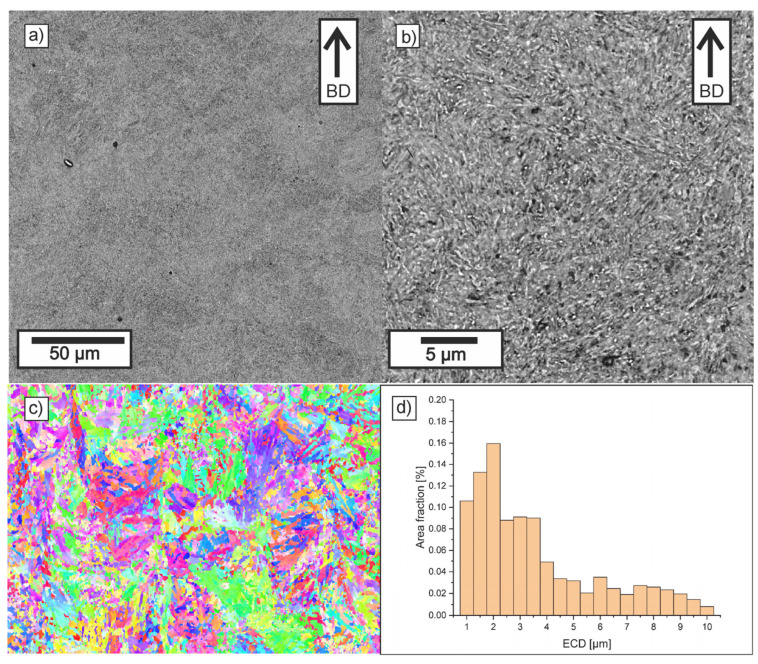
Microstructure of Sample 11: (**a**,**b**) SEM (BD-build direction), (**c**) EBSD IPF, and (**d**) grain size distribution.

**Figure 10:**
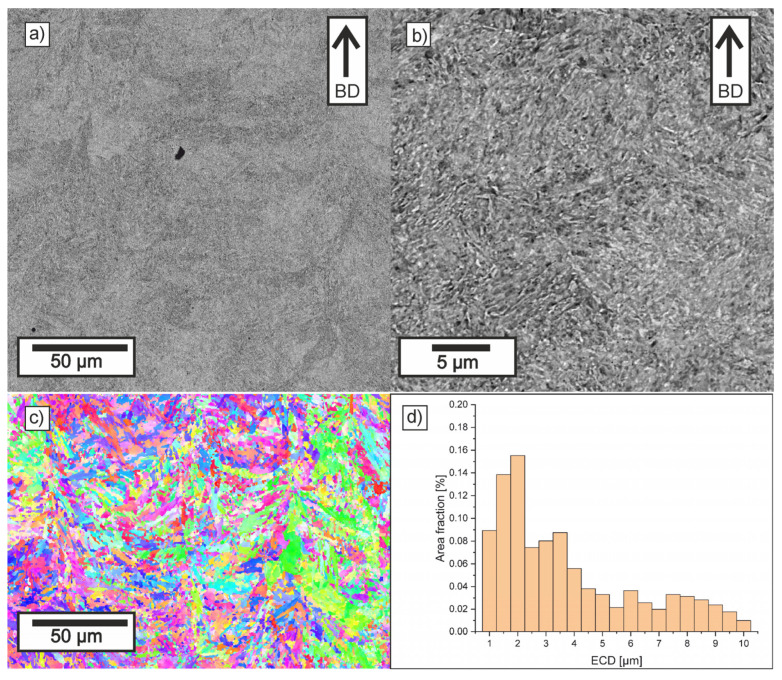
Microstructure of Sample 29: (**a**,**b**) SEM (BD-build direction), (**c**) EBSD IPF, and (**d**) grain size distribution.

**Figure 11:**
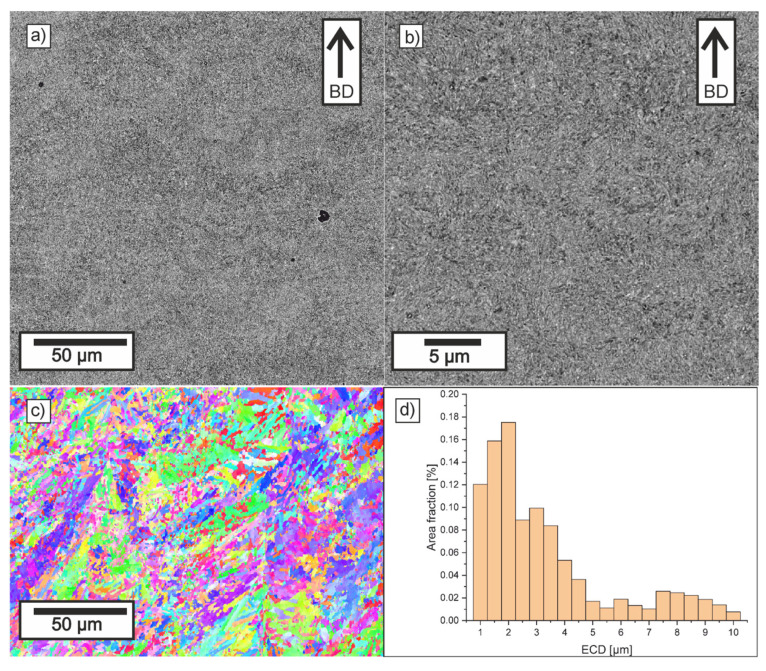
Microstructure of Sample 30: (**a**,**b**) SEM (BD-build direction), (**c**) EBSD IPF, and (**d**) grain size distribution.

Figure 8, Figure 9, Figure 10 and Figure 11 show the microstructure observed in SEM images, EBSD maps, and grain-size histograms for the four parameter variants. The SEM observations revealed that all materials exhibited a fine-grained microstructure, resulting from rapid cooling during the LPBF process. The microstructure is dominated by tempered martensite. All samples contain crystallized melt pools, with visible differences in grain size between their boundaries and interiors.

Higher cooling rates occur at melt-pool boundaries, where the rapid solidification leads to the formation of cyclic zones with ultra-fine grains. In contrast, the interiors of melt pools solidify more slowly, which promotes the formation of elongated primary grains aligned with the heat-flow direction. This behavior is consistent with trends reported for low-alloy steels processed by LPBF.

Within the tempered-martensite matrix, submicron inclusions with darker contrast are visible, likely corresponding to oxide dispersions. Although the detailed mechanisms of oxide formation in LPBF steels are discussed in the literature, in this study, only fine, uniformly distributed inclusions were observed, and no coarse oxides that could reduce mechanical strength were detected. The area fraction of oxides was similar in all parameter variants.

The microstructure near the melt-pool boundaries is more pronounced for lower hatch distances, whereas higher laser energy density leads to deeper melt pools and stronger temperature gradients in adjacent layers, promoting more intense tempering of martensite. These tendencies align with general observations reported in previous studies on LPBF-processed low-alloy steels.

Compared to samples 11 and 30, the LPBF specimens made with parameters 10 and 29 showed melt pools that were notably deeper and narrower. The ECD distributions for all investigated samples were similar, with grains in the range 0.5–15 µm, with more than 50% of the area consisting of grains with ECD smaller than 2.5 µm. It can be observed that in Sample 11, there is a slight reduction in the area fraction of small grains and an increase in the area fraction of grains larger than 6 µm. It was confirmed that in deep keyhole mode, solidifying grains from both sides of the deep keyhole effectively stops the growth of grains from the bottom of the track [63,64]. Thus, despite the higher VED, the microstructure formed is slightly finer than in Sample 10.

For variants manufactured at a larger hatch distance, the grain size relationship is correlated with VED (LED), and a slightly lower area fraction of the smallest grains and a higher area fraction of coarser grains are observed in variant 29. In the cases of increased hatch distance, the width of the crystallized melt pools was significantly wider, which allowed for less competitive grain growth.

The EBSD maps provided additional insight into the crystallographic structure and grain-size distribution in the analyzed specimens. All parameter variants exhibited a heterogeneous mixture of fine and elongated grains, reflecting the combined effects of melt-pool geometry and remelting between the adjacent tracks and layers. The observed fine grain structure was formed as a result of rapid cooling and the transformation of austenite grains into martensite. The differences between the variants arise mainly from the initial geometry of melt pools obtained and from the fraction of the parts that remain after the subsequent layer is produced. It is worth emphasizing that the observed differences arise directly from the cooling rate implied by the applied LPBF parameters—scan speed, and hatch distance—which determine the size of melt pool and solidification dynamics.

The EBSD orientation maps revealed subtle differences between the samples. Specimens processed at lower VED values (especially sample 30) showed a more homogeneous distribution of orientations and a higher fraction of equiaxed grains, indicating fast solidification conditions and a high cooling rate characteristic of lower VED parameters. In contrast, sample 11 exhibited a slightly broader spread of orientations and a higher fraction of coarse grains, which is consistent with shallower melt pools resulting from lower laser power and higher scanning speed.

Overall, the SEM and EBSD results confirm that the microstructural formation in LPBF-processed low-alloy steels is strongly governed by the cooling rate associated with VED and size of melt pool. Lower VED produced the most homogeneous and fine-grained microstructure of martensite (sample 30), whereas insufficient energy input (sample 11) resulted in coarser grains.

### 3.3. Impact of VED and Printing Orientation on Bending Strength of 3D-Printed 25CrMo4 Steel

The specimens were manufactured in a vertical build orientation, while the three-point bending tests were performed perpendicular to the build layers. The results, summarized in Table 6, reveal a strong correlation between the volumetric energy density (VED) and bending strength. (Table 6).

Sample 10, fabricated with the highest VED (89.3 J/mm^3^), exhibited the lowest strength (2587.8 MPa), likely due to excessive heat input causing formation of higher amount of tempered martensite. Sample 11 (71.4 J/mm^3^) reached 2878.6 MPa, representing a balanced process condition. The highest strength was obtained for sample 29 (4004.6 MPa) at 66.7 J/mm^3^, suggesting a favorable compromise between the density and microstructure. Sample 30, produced with the lowest VED (55.6 J/mm^3^), also achieved a high strength of 3285.4 MPa, indicating that reduced energy input—when appropriately selected—can still ensure efficient properties.

The obtained results indicate that bending performance in LPBF-processed 25CrMo4 steel can be tailored by hatch distance adjustment. For samples with higher hatch distance, the increase in bending strength is related to lower heat accumulation in samples.

### 3.4. Mechanical Response of Compressed Samples at Different VED Values

**Figure 12:**
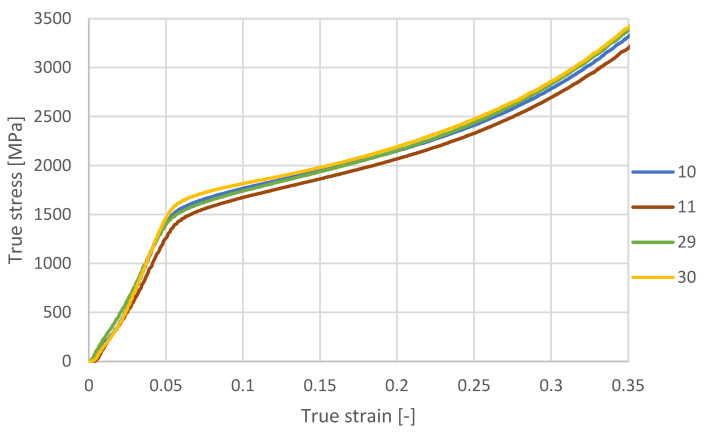
True strain for compressed samples at different VED values.

The chart in Figure 12 shows the correlation between true stress and true strain for four samples subjected to a compression test at different VED values.

Sample 30, which reached the highest true stress (~3400 MPa), confirms that appropriately controlled solidification conditions—even at the lowest VED used in this study (55.6 J/mm^3^)—can provide excellent mechanical performance. This observation is consistent with findings reported for AISI 4130 and 3130 steels, where optimized processing parameters enabled strong interlayer bonding and high load-bearing capability without the need for maximizing energy input [62,63,64]. Sample 29 (LED = 0.18 J/mm, VED = 66.7 J/mm^3^) exhibits an intermediate mechanical response. Its stress values are higher than those of sample 11 but remain slightly lower than those of samples 10 and 30, and its true stress–true strain curve progresses smoothly between the highest and lowest curves throughout the test. The behavior of sample 29 corresponds to its moderate energy input, which positions this specimen between the low- and high-energy regimes observed in the remaining samples. In contrast, sample 11 (LED = 0.15 J/mm, VED = 71.4 J/mm^3^), which recorded the lowest true stress (~3150 MPa), reflects the adverse effects of insufficient energy input. Its curve remains below the other samples across the entire strain range, consistent with the defects and reduced melt-pool stability typically associated with low-energy LPBF conditions [65]. Sample 10, despite being processed with the highest VED (89.3 J/mm^3^), does not outperform sample 30. Its lower bending strength reported earlier is likely related to excessive energy input, which can promote overheating or local microstructural softening, in agreement with trends described in the literature [49]. Overall, the results show that mechanical strength in this study did not increase monotonically with rising VED. Instead, the highest performance was obtained at the lowest VED value (sample 30), while both insufficient energy (sample 11) and excessive energy (sample 10) led to reduced strength. This indicates that, for the investigated low-alloy steel, the processing window lies in the lower VED range, where melt-pool stability is achieved without overheating. These findings align with recent LPBF research emphasizing that precise tuning of processing parameters—rather than maximizing energy input—is essential for controlling melt-pool behavior and achieving application-specific mechanical properties [61,64,65].

### 3.5. Hardness Measurements

Table 7 summarizes the hardness results, which are consistent with microstructural observations. Sample 10 exhibited the most uniform hardness distribution, attributed to a higher fraction of fine grains in the keyhole region and more consistent tempering at high VED. In contrast, Sample 11 exhibited shallow melt pools and a mixed fine/coarse grain structure, resulting from a lower energy input and less uniform tempering. Larger hatch distances further increased melt pool size and hardness variations, consistent with findings in LPBF steels [61,66].

Similar cyclic hardness fluctuations were observed in the present study, with higher hardness values occurring at the melt-pool boundaries where finer grains were present. A comparable trend was reported in the literature, where authors also associated increased hardness with the fine grain microstructure at melt-pool boundaries. The decrease in hardness and the reduced coefficient of variation (CV) at higher VED values observed in our results may be attributed to partial in situ tempering caused by the additional heat input during subsequent laser passes. A similar mechanism, where increased energy input promotes spontaneous tempering, has been described in the literature [67]. A tendency towards more homogeneous microstructure formation at lower hatch distances was also evident in this work. Under these conditions, the material exhibited finer and more uniformly oriented grains. This observation is consistent with findings reported for other alloys processed by LPBF, including NiTi and Ni-based superalloys, where reduced hatch spacing led to more uniform thermal gradients and grain refinement [64,68].

### 3.6. Stress–Strain Test

**Figure 13:**
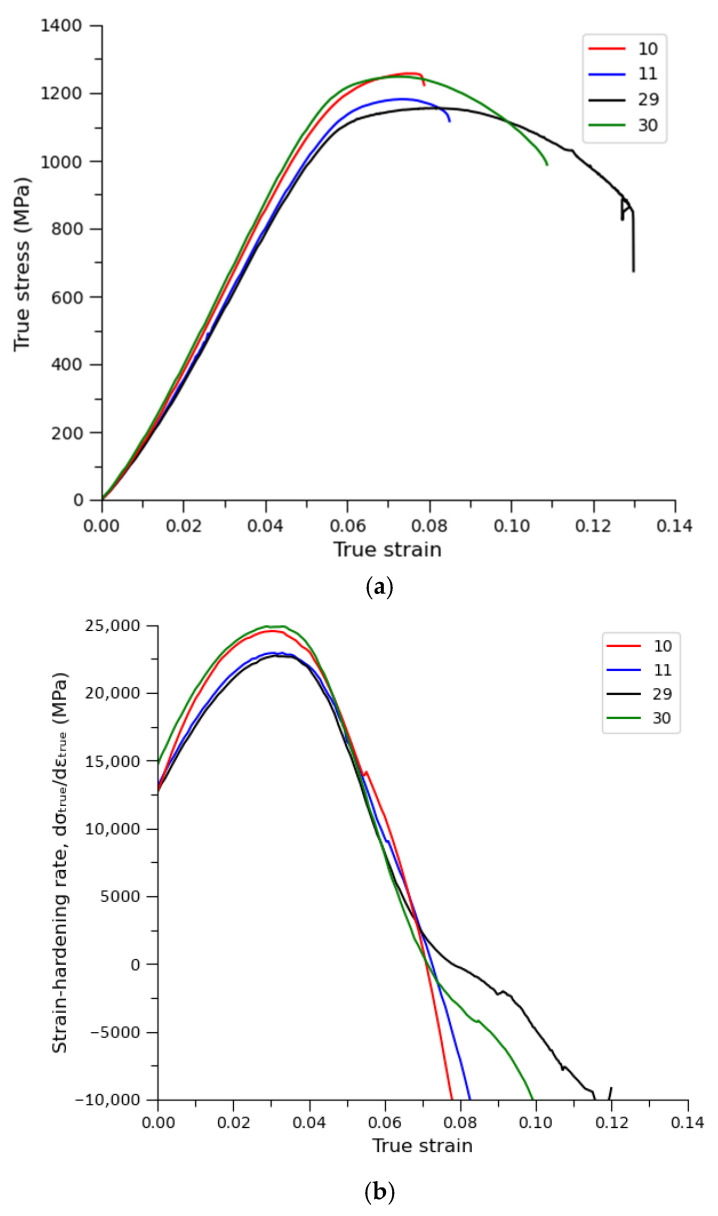
(**a**) Tensile stress–strain curves (**b**) strain-hardening for selected 3D printed 25CrMo4 steel samples.

Figure 13 presents the representative stress–strain curves for the examined 25CrMo4 steel specimen produced by LPBF and clearly highlights the differences in mechanical response between the four parameter variants. The curves exhibit the typical profile of ductile metallic materials, including a linear elastic region, followed by smooth plastic deformation and a final segment corresponding to the onset of necking.

The tensile test results show distinct differences depending on the processing parameters. Sample 30 achieved the highest ultimate tensile strength (~1250 MPa) with stable plastic flow, which is consistent with the fine and homogeneous microstructure obtained at the lowest VED (55.6 J/mm^3^). Sample 10 reached a comparable strength (~1230 Mpa) but exhibited earlier necking, which may indicate localized softening caused by excessive thermal input (VED = 89.3 J/mm^3^). Sample 29 showed an intermediate strength level (~1190 Mpa) and a stable deformation profile, whereas sample 11 achieved the lowest strength (~1150 Mpa), likely due to microstructural heterogeneity and partial grain coarsening resulting from insufficient energy input.

Table 8 summarizes the key mechanical properties obtained from tensile tests for the four investigated LPBF-processed 25CrMo4 steel samples. The yield strength and necking stress were determined for a single representative specimen from each parameter set, whereas the UTS values were measured five times, enabling the calculation of the mean and standard deviation. The compiled data allow for a direct comparison of the influence of processing parameters on strength and result variability.

Based on the obtained values, it can be observed that the highest strength parameters were achieved by the two extreme samples—sample 10 (processed with the highest VED) and sample 30 (processed with the lowest VED). Despite exhibiting similar UTS levels, these samples show different deformation behavior: sample 10 is characterized by earlier necking, while sample 30 demonstrates a more stable plastic-flow response. The intermediate samples (particularly 29 and 11) show lower strength values, indicating that both insufficient and excessive energy input may negatively affect the microstructure and mechanical performance. The low standard deviations of UTS confirm good measurement repeatability and process stability within the analyzed parameter range. 

The mechanical behavior observed in this study is also consistent with recent findings reported for steels and alloys processed by LPBF. Numerous publications indicate that both excessively low and excessively high VED values lead to increased porosity, melt-pool instability, and deterioration of mechanical properties, while the most favorable properties are achieved within a narrow, well-controlled processing window rather than at maximum energy input. These relationships have been demonstrated for low-alloy steels, stainless steels, and nickel-based alloys [66,67,68,69,70], and further supported by recent studies on the development of steels dedicated to LPBF processing [56,71]. The trends identified here for 25CrMo4 steel therefore align with the broader understanding that it is energy optimization—rather than maximization—that governs the formation of a homogeneous microstructure and high mechanical performance in LPBF-manufactured components.

## 4. Conclusions

This study demonstrated that the mechanical performance and microstructural integrity of 25CrMo4 (AISI 4130) steel produced via laser powder bed fusion (LPBF) are highly dependent on the selection of process parameters, particularly volumetric and linear energy density (VED and LED). The results confirmed that excessive energy input, as observed for sample 10 with the highest VED (~89.3 J/mm^3^), led to deteriorated mechanical behavior due to residual stresses and possible microcrack formation, despite achieving fine-grained martensitic structures.

Conversely, samples processed at moderate energy input levels (samples 29 and 30, VED~55–67 J/mm^3^) exhibited superior mechanical properties, including higher bending and tensile strength, improved ductility, and a more stable compressive response. These results indicate that increasing the energy input beyond this range does not necessarily improve performance; instead, a balanced and well-controlled set of parameters yields the most favorable outcomes.

Microstructural analyses confirmed that moderate VED values promote uniform melting, reduced porosity, and homogeneous grain structure, while higher VED values increase the risk of keyhole formation and localized thermal defects. The manual porosity assessment supported this conclusion, with lower porosity levels observed in the best-performing samples (10 and 11) compared to those produced under more extreme conditions (29 and 30). Importantly, fine oxide inclusions were detected in all specimens; however, they did not negatively impact the strength, suggesting that the LPBF process window allows for stable inclusion control in this steel grade.

The innovative aspect of this study stems from its examination of 25CrMo4 steel, a heat-treatable low-alloy steel with broad industrial relevance, yet one that remains underrepresented in current additive manufacturing research. While alloys such as Ti6Al4V and 316L stainless steel dominate the AM literature, systematic investigations on 25CrMo4 are virtually absent. The present work, therefore, fills a significant knowledge gap by providing a comprehensive assessment of how LPBF parameters affect the microstructure, porosity, and mechanical behavior of this material. The identification of a process window (VED~55–70 J/mm^3^) constitutes a valuable contribution to both science and industry, enabling the design of reliable, lightweight, and mechanically robust components from a material previously regarded as challenging for AM.

The ability to achieve high strength, good ductility, and controlled porosity through careful adjustment of process parameters opens up new opportunities for replacing conventionally manufactured parts with additively manufactured counterparts, particularly in designs that require geometric complexity, weight reduction, and superior mechanical performance.

In conclusion, this research provides experimental evidence that moderate and well-controlled energy input maximizes the performance of 25CrMo4 steel in LPBF and establishes a scientific basis for the further development of process guidelines for heat-treatable steels in additive manufacturing.

## Figures and Tables

**Table 1:** Chemical composition of the 25CrMo4 powder, as provided by the manufacturer.

Element	Percentage Composition [%]
Cr	0.70–1.20
Mn	0.30–0.70
Mo	0.10–0.40
Si	0.20–0.50
C	0.27–0.34
P	≤0.03
S	≤0.04
Fe	Balance

**Table 2:** The parameters of the printing material.

No.	Laser Power [W]	Scanning Speed [mm/s]	Hatch Distance [mm]	Layer Thickness [mm]	LED [J/mm]	VED [J/mm^3^]
1	90	800	0.07	0.03	0.112	53.6
2	90	1000	0.07	0.03	0.090	42.9
3	90	1200	0.07	0.03	0.075	35.7
4	110	800	0.07	0.03	0.137	65.5
5	110	1000	0.07	0.03	0.110	52.4
6	110	1200	0.07	0.03	0.092	43.6
7	130	800	0.07	0.03	0.162	77.4
8	130	1000	0.07	0.03	0.130	61.9
9	130	1200	0.07	0.03	0.108	51.6
10	150	800	0.07	0.03	0.187	89.3
11	150	1000	0.07	0.03	0.150	71.4
12	150	1200	0.07	0.03	0.125	59.5
13	170	800	0.07	0.03	0.212	101.2
14	170	1000	0.07	0.03	0.170	80.9
15	170	1200	0.07	0.03	0.142	67.5
16	100	800	0.09	0.03	0.125	46.3
17	100	1000	0.09	0.03	0.100	37.0
18	100	1200	0.09	0.03	0.083	30.9
19	120	800	0.09	0.03	0.150	55.6
20	120	1000	0.09	0.03	0.120	44.4
21	120	1200	0.09	0.03	0.100	37.0
22	140	800	0.09	0.03	0.175	64.8
23	140	1000	0.09	0.03	0.140	51.8
24	140	1200	0.09	0.03	0.117	43.2
25	160	800	0.09	0.03	0.200	74.1
26	160	1000	0.09	0.03	0.160	59.3
27	160	1200	0.09	0.03	0.133	49.4
28	180	800	0.09	0.03	0.225	83.3
29	180	1000	0.09	0.03	0.180	66.7
30	180	1200	0.09	0.03	0.150	55.6

**Table 3:** Porosity and melting modes depending on laser power and scanning speed (hatch distance: 70 µm, layer thickness: 30 µm).

Laser Power [W]	Laser Scanning Speed [mm/s]		
	800	1000	1200
90	1 (lack of fusion)	2 (lack of fusion)	3 (lack of fusion)
110	4 (process window)	5 (lack of fusion)	6 (lack of fusion)
130	7 (process window)	8 (slight lack of fusion)	9 (small lack of fusion)
150	10 (small keyhole)	11 (process window)	12 (slight lack of fusion)
170	13 (keyhole)	14 (small keyhole)	15 (process window)

**Table 4:** Porosity and melting modes depending on laser power and scanning speed (hatch distance: 90 µm, layer thickness: 30 µm).

Laser Power [W]	Laser Scanning Speed [mm/s]		
	800	1000	1200
100	16 (lack of fusion)	17 (lack of fusion)	18 (lack of fusion)
120	19 (slight lack of fusion)	20 (lack of fusion)	21 (lack of fusion)
140	22 (process window)	23 (slight lack of fusion)	24 (slight lack of fusion)
160	25 (small keyhole)	26 (process window)	27 (slight lack of fusion)
180	28 (small keyhole)	29 (small keyhole)	30 (process window)

**Table 5:** Porosity of 25CrMo4 steel samples (%).

Measurement	Sample 10	Sample 11	Sample 29	Sample 30
Average porosity, %	0.11	0.35	0.21	0.25

**Table 6:** Three-point bending test results—strength and strain energy.

Sample	Bending Strength [MPa]	LED [J/mm]	VED [J/mm^3^]
10	2587.8	0.18	89.3
11	2878.6	0.15	71.4
29	4004.6	0.18	66.7
30	3285.4	0.15	55.6

**Table 7:** Results of hardness measurements, average hardness with standard deviation, hardness range, and coefficient of variation.

Sample	Hardness Range [HV1]	Coefficient of Variation [CV] [%]	Average Hardness [HV1]	Standard Deviation [HV1]
10	456–476	1.7	467	8
11	427–464	3.6	442	16
29	430–474	3.3	447	15
30	408–506	6.4	466	30

**Table 8:** Summary of tensile test results for the investigated samples.

Sample	Yield Strength [MPa]	Necking [MPa]	Average UTS [MPa]	Standard Deviation of UTS [MPa]
10	993	1250	1342.7	27.8
11	928	1250	1297.7	30.3
29	911	1142	1248.2	33.9
30	967	1181	1324.9	38.63

## Data Availability

The original contributions presented in this study are included in the article. Further inquiries can be directed to the corresponding authors.
